# Scoping review on the use of South-South learning exchange to scale up evidence-based practices in family planning

**DOI:** 10.1136/bmjgh-2022-011635

**Published:** 2023-06-12

**Authors:** Komal Preet Allagh, Isotta Triulzi, James Kiarie, Rita Kabra

**Affiliations:** 1 Consultant- Department of Sexual and Reproductive Health and Research, World Health Organization, Geneva, Switzerland; 2 Institute of Management, Scuola Superiore Sant'Anna, Pisa, Italy; 3 Department of Sexual and Reproductive Health and Research including UNDP/UNFPA/UNICEF/WHO/World Bank Special Programme of Research, Development and Research Training in Human Reproduction, World Health Organization, Geneva, Switzerland

**Keywords:** Review

## Abstract

**Background:**

South-South learning exchange (SSLE) is an interactive learning process where teams from low-income and middle-income countries exchange knowledge and experience to support one or both team’s work towards a change in policies, programmes or practices. SSLE has been used by countries to improve family planning (FP) outcomes such as increased contraceptive prevalence rate and reduced unmet need for FP, but at present, there are no reviews that summarise its use. We conducted a scoping review with stakeholder consultations to summarise the use of SSLE to change FP outcomes.

**Objective:**

To systematically identify and map the purposes, approaches, outputs, outcomes, enablers and barriers to using SSLE in FP.

**Methods:**

A search was conducted on electronic databases, grey literature sources, websites and the reference list of included studies. The scoping review is based on an adapted version of Arksey and O’Malley’s scoping review framework suggested by Levac *et al*. Experts were interviewed on their experiences in SSLE.

**Results:**

The initial search yielded 1483 articles; however, only 29 were selected in the final analysis. The articles were published between 2008 and 2022. Most of the articles were reports, case studies or press releases, only two were peer-reviewed publications. Capacity building of FP providers, policy-makers and community was the most commonly reported purpose of SSLE, with study tours (57%) being the most common approach. Policy dialogue was the most common (45%) output and improved contraceptive prevalence was the most frequently reported outcome. The experiences of the 16 interviewed experts aligned with the scoping review findings.

**Conclusion:**

The evidence on the effectiveness of SSLE for addressing FP outcomes is very limited and of very low quality. We call on stakeholders conducting SSLE to document their experiences in detail, including the outcomes achieved.

**WHAT IS ALREADY KNOWN ON THIS TOPIC**

- South-South learning exchanges (SSLEs) have been used between countries to share knowledge and experiences on family planning (FP).

**WHAT THIS STUDY ADDS**

- To the best of our knowledge, this study is the first evidence synthesis on this topic. Only 29 articles on SSLE in FP (process, approach, output, outcome, enablers and barriers) were found. Of these, only two were peer-reviewed publications.
- Sixteen experts with experience in conducting SSLE in FP and in other areas were interviewed using a semistructured questionnaire. They identified SSLE as an important process for knowledge exchange but highlighted the need for a standard methodology to conduct SSLE.
- Capacity building of FP providers, policy-makers and community was the most common purpose of SSLE; study tours were the most common approach used, and policy dialogue was the most frequent output from SSLE.

**HOW THIS STUDY MIGHT AFFECT RESEARCH, PRACTICE OR POLICY**

- There is a need for more research on determining the extent to which SSLE has contributed to FP outcomes.
- Further studies are needed to identify the best approach to conduct SSLE to improve access to right-based equitable FP services.
- There is a need to document the extent to which SSLE has contributed to FP outcomes.

## Introduction

Currently, there is no standard definition for South-South learning exchange (SSLE). Sometimes referred to as peer-to-peer learning exchange, South-South cooperation (SSC) or knowledge exchange. For this review, we will use a working definition as identified in the WHO guidance document^1^; SSLE is an interactive learning process where stakeholder teams from low-income and middle-income countries exchange knowledge and experience to support one or both teams working towards a change by identifying, adopting and/or strengthening the implementation of a best practice.^1^ SSLE can be used for the following purposes: to identify/share good practices, knowledge management, advocate policy or programme change or capacity building.^1^ These learning exchanges can generate new ideas, knowledge and approaches that work, inspire leaders to implement reforms/policy dialogue, exchange practical ‘how-to’ knowledge for solving problems, and foster collaboration and capacity building of participants to advocate for more effective change processes or best practices. By sharing new knowledge and skills between programme managers and policy-makers from geographically distinct locations but with similar contexts and facing similar challenges, SSLE is often far more convincing and contextually appropriate than learning from publications or experts.^2^

SSLE can occur between governments, non-government organisations, private sector, community-based organisations and civil society organisations across states, regions or countries.^3^ In the past, learning exchanges have focused on a wide range of topics, from trade, finance, food security, nutrition and health.^3^

In 2019, WHO embarked on the Family Planning Accelerator project^4^ to improve access to quality and rights-based family planning (FP) services. Under this project, SSLE is used to facilitate the implementation and scaling up of evidence-based practices for the uptake of postpartum and postabortion FP (PPFP) services and to expand the range and choice of contraceptives available.

Several countries have reported using SSLE to improve FP outcomes, such as increased contraceptive prevalence rate and reduced unmet need for FP. To our knowledge, no published review summarises the uses and outcomes of SSLE in FP. In this paper, we report the findings of a scoping review on the purposes, approaches, barriers, enablers and outcomes of SSLE in FP.

We systematically reviewed the entire range of published and grey literature on SSLE in FP. The impetus for this review arose from the need to find innovative, evidence-based solutions to enhance the implementation and scale-up of evidence-based practices. Therefore, by carrying out this scoping review, we want to bring to the attention of programme managers and policy-makers the best evidence-based practices to improve knowledge sharing and support informed decisions to scale up evidence-based practices in FP.

## Methods

This scoping review is based on the adapted version of Arksey and O’Malley’s scoping review framework suggested by Levac *et al*.^5 6^ We are reporting the study as per the Preferred Reporting Items for Systematic Reviews and Meta-Analyses Scoping Reviews (PRISMA-ScR) reporting guidelines and this checklist is provided in online supplemental material 1.

10.1136/bmjgh-2022-011635.supp1 Supplementary data

### Step 1: identification of research questions

This scoping review was conducted to answer the following research questions:

1. For what purposes has SSLE in FP been used?
2. What approaches and methods have been used to conduct SSLE in FP?
3. What are the barriers and enablers encountered in conducting SSLE in FP?
4. What outcomes have been achieved by SSLE in FP?

### Step 2: identification of relevant studies—search strategy

To address the research questions, we developed a search strategy in consultation with the chief librarian at WHO to identify all relevant publications on SSLE in family planning. The following search terms were identified and used: *South-South learning exchange*, *South-South knowledge exchange*, *South-South exchange*, *Peer to peer learning exchange*, *South-South cooperation*, *information sharing*, *information exchange*, *knowledge sharing*, *knowledge exchange*, *learning exchange*, *Family planning*, *contraception*, *reproductive health*. Using the search terms, we systematically searched the following electronic databases: MEDLINE, CINAHL, Embase, Hinari, ProQuest DB, PubMed, Web of Science, WorldCat and Google Scholar for published articles up to 23 July 2021. The full electronic search strategy is included in online supplemental file 2. No limits were placed on the searches.

10.1136/bmjgh-2022-011635.supp2 Supplementary data

An example of the search strategy in Embase is: (‘South-South’ OR (‘developing country’/exp AND ‘international cooperation’/exp/mj)) AND (‘learning exchange’ OR ‘knowledge exchange’ OR ‘Peer to peer’ OR ‘cooperation’ OR ‘information sharing’ OR ‘information exchange’ OR ‘knowledge sharing’ OR ‘knowledge exchange’ OR ‘learning exchange’) AND (reproduct* OR ‘family planning’ OR contracept* OR ‘Family Planning Services’ OR ‘Reproductive Medicine’ OR ‘Population Control’ OR ‘Population Growth’ OR Contraception OR Fertility OR ‘Contraception Behavior’ OR ‘Embryo Transfer’ OR ‘Intrauterine Devices’ OR ‘Long Acting Reversible Contraception’ OR ‘Maternal Child Health Centers’ OR ‘Pregnancy In Adolescence’ OR ‘Reproductive Techniques Assisted’)

Furthermore, websites such as, FP 2030 partners in population and development (PPD), UNFPA (United Nations Population Fund), WHO, UNOSCC (United Nations Office of South-South cooperation), South-South Galaxy, World Bank, UNDP (United Nations Development Programme) and United States Agency for International Development were searched for scientific reports and papers on SSLE in FP. In addition, we searched conference proceedings and student thesis on SSLE in FP. We also searched the reference lists of all the included studies to identify any relevant additional studies, and during stakeholder interviews, we asked the experts to share relevant documents. The search was most recently rerun on 29 August 2022 (two further articles were included).

### Step 3: study selection

An eligibility criterion was established to ensure that the included studies contain the specific information to answer the research questions and objectives of this scoping review. We only included studies using SSLE between countries to improve FP outcomes. We excluded studies using SSLE between districts or states, or organisations because our focus was on learning exchanges at the Ministry of Health (Central government) level and not at the facility level. No limits were placed on the study period, language and study location. The eligibility criteria are summarised in table 1.

**Table 1:** Inclusion and exclusion criteria

Criteria	Inclusion	Exclusion
Location	Any country	None
Date	Any year	None
Language	All languages	None
Focus area	Studies focusing on objectives, purpose, approaches, process, enablers, barriers and outcomes of SSLE in family planning	SSLE on topics other than family planning.
Geographical and administrative jurisdiction	Learning exchanges between countries	Learning exchange is within country (between institutions, cities or districts)
Document type	Scientific reports, case study, commentaries, research articles, conference proceedings, student thesis, letters to editors and reviews	Newspaper, power point presentations and magazine articles

SSLE, South-South learning exchange.

After running the search, all the retrieved studies were exported into Zotero V.5.0.96.2 reference management software. We used a two-part study selection process (relevance screening). First, two independent reviewers (KPA and RK) screened the title and abstract of all the retrieved citations for inclusion guided by the eligibility criteria. Any article considered relevant by either or both reviewers was included for full-text review. In the second step, the reviewers independently assessed the full-text articles to determine if they met the inclusion criteria. Where differences arose, the reviewers consulted a third reviewer (JK) to reach a consensus. The literature identified from the scoping review was used to create a narrative.

### Step 4: data charting

A data-charting form was prepared in Microsoft excel to extract characteristics of included studies and was constantly updated through the review process. We extracted the following data from the included studies: (1) general characteristics: authors, title, year and source of publication, countries involved in SSLE, number of participants; (2) purpose/focus of SSLE, duration of SSLE, key stakeholders; (3) methods used/ approach for SSLE, the process; (4) barriers, enablers of SSLE and (5) FP outputs and outcomes.

### Step 5: collating, summarising and reporting data

The extracted evidence was repeatedly reviewed to summarise into a narrative. All authors reviewed the extracted information to summarise the findings across the articles. Since the research questions were broad, the results of the review were synthesised thematically to report on: the purpose, methods and processes, outputs and outcomes and the barriers and enablers of SSLE in FP.

### Step 6: stakeholder consultations

The consultations used a qualitative study design (key informant interviews) to understand the stakeholder’s perceptions and experiences of applying SSLE in FP. Experts from UNFPA, WHO, PPD and the government were conveniently selected for the in-depth interviews. The participants were assured of the confidentiality of the interviews. All interviews were conducted virtually via Google meet and were recorded using a voice recorder and by taking notes. Each interview took 30–45 min.

Each participant was provided with an overview of the scoping review. A semistructured interview guide was developed. This questionnaire included a set of open-ended questions to guide the discussion and covered the following topics: (1) role of the organisation in SSLE and the process used, (2) purpose of SSLE, (3) views and experiences on SSLE in FP (4) perception on challenges and successes observed during the SSLE process and (5) lessons learnt. All the interviews were transcribed verbatim. After reviewing all the transcripts, a codebook was developed, piloted on two transcripts and finalised. Data were imported into R-based Qualitative Data analysis vV.0.2-8, which supports coding and data management. Transcripts were reviewed line by line and assigned codes.

The data were narratively summarised according to predefined themes on the approaches used for SSLE, the purpose of SSLE, key FP outputs, outcomes, barriers and enablers of SSLE and lessons learnt. Based on the scoping review results, these themes were developed following discussion among authors. Synthesis from the review was integrated with the stakeholder’s consultation.

As mentioned earlier, these stakeholder consultations were essential to understand and gather first-hand information on the use of SSLE in FP.

The scoping review qualifies for an exemption based on the Council for International Organizations of Medical Sciences criteria and the WHO ERC RoP since: ‘(1) it does not involve human participants’ and ‘(2) public officials are interviewed in their official capacity on issues that are in the public domain’.

### Patient and public involvement

Patients and the public were not involved in the design, conduct, reporting or dissemination plans of our scoping review.

## Results

### Study inclusion

**Figure 1:**
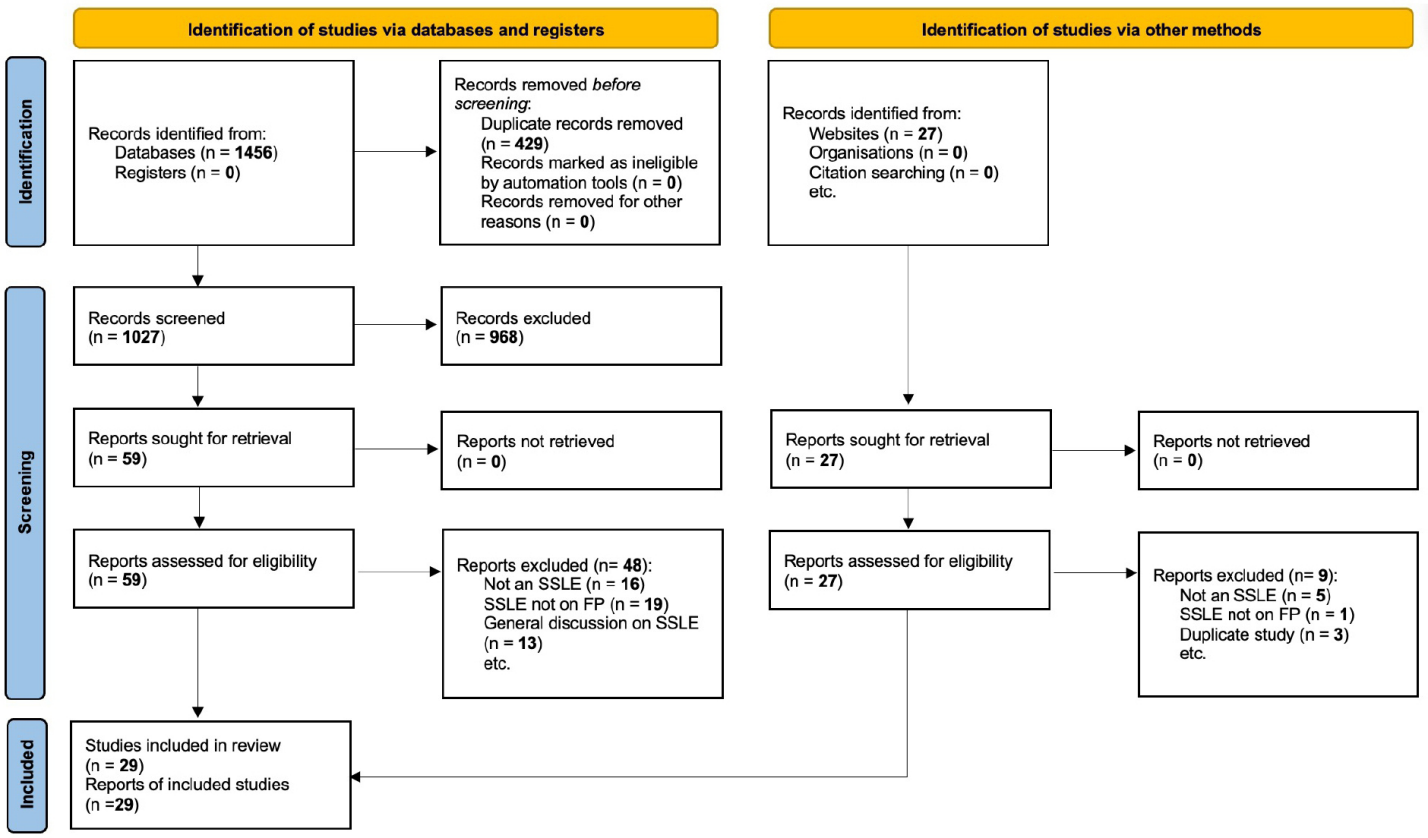
Preferred Reporting Items for Systematic Reviews and Meta-Analyses flow chart. FP, family planning; SSLE, South-South learning exchange.

The initial search yielded 1456 articles from electronic databases and 27 from manual searching. Of these, 86 articles were included for full-text review. A total of 29 articles were included in the final analysis. Reasons for exclusion are provided in the PRISMA chart (figure 1).

### Characteristics of included studies

The included articles included 19 case study reports (65.5%), 8 press releases (27.6%) and 2 scientific manuscripts (6.9%). The articles were published between 2009 and 2022, with 79% published between 2018 and 2022. All included articles were published in the English language. The studies reported SSLE in FP from 58 countries.

Source of publication: Of the 29 articles, 27 were published in organisational reports: 10 (34.5%) were published by UNFPA, 5 (17%) by the National Population and FP board (Badan Kependudukan dan Keluarga Berencana Nasional-BKKBN) Indonesia, 5 (17%) by UNOSCC, 2 (7%) by Indonesia’s National coordination team of South-South and Triangular Cooperation, 1 (3.4%) by World bank, 1 (3.4%) by FP2020/WHO, 1 (3.4%) by Department of Health, Philippines, 1 (3.4%) by UNDP and 1 (3.4%) by FHI 360. Only two were in the peer-reviewed journals: one in *Global Health*: Science and Practice and one in *BMJ Global Health*. Table 2 depicts the general characteristics of the studies and reports.

**Table 2:** Summary of general characteristics of included studies

S. no	Title (ref)	Countries	Type of document	Year published	Year of SSLE	Source of publication	Objectives reported				
							**Purpose**	**Approach**	**Output**	**Outcome**	**Enablers**
South-South and Triangular Cooperation (SSTC) in Action: Sexual and Reproductive health^18^											
1.	Cross-cultural partnerships with Muslim religious leaders for FP (Case study) (18a)	Afghanistan, Algeria, Azerbaijan, Bangladesh, Burundi, Chad, Ethiopia, Ghana, Guinea, India, Malaysia, Maldives, Mali, Nepal, Nigeria, Pakistan, Philippines, Sudan, Sri Lanka, Indonesia	Report (case study)	2018	2013–2018	UNFPA	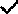	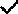	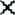	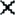	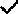
2.	Cooperation in FP and women’s role in a Muslim setting (case study) (18b).	Philippines, Indonesia	Report (case study)	2018	2012–2017	UNFPA	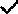	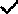	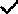	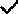	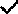
3.	Promotion of FP by Muslim religious leaders (case study) (18c).	Indonesia, Bangladesh, Egypt, Morocco, Chad	Report (case study)	2018	2017	UNFPA	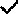	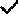	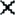	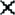	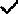
4.	Involving men in the promotion of reproductive health through schools for husbands (case study) (18d).	Burkina Faso, Cameroon, Côte D’Ivoire, Guinea, Mali, Mauritania, Senegal, Sierra Leone, Togo, Niger	Report (case study)	2018	2019	UNFPA	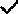	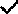	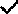	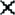	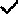
5.	Regional training centre of excellence (case study) (18e).	Comoros, Eswatini, Lesotho, Madagascar, Namibia, Seychelles, Zambia, Mauritius	Report (case study)	2018	2015	UNFPA	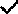	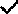	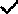	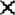	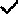
6.	Upgrading midwifery education programme and services (case study) (18f).	Sudan, Morocco	Report (case study)	2018	2017	UNFPA	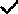	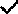	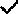	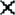	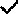
South-South Cooperation as a mode of engagement: Innovative programme solutions^22^											
7.	Centre of Excellence on Unmet need for FP (case study) (22a).	Afghanistan, Bangladesh, Egypt, Ghana, Lao People’s Democratic Republic, Myanmar, Pakistan, Papua New Guinea, Sri Lanka, Timor-Leste, Indonesia	Report (case study)	2021	2014–2021	UNFPA	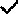	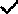	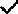	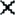	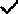
8.	Muslim Religious leaders engage in local FP Actions (case study) (22b).	Nepal, Indonesia	Report (case study)	2021	2016–2020	UNFPA	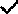	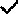	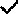	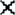	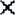
9.	Maternal health services in the midst of a measles outbreak (case study) (22c).	Samoa, Fiji	Report (case study)	2021	2019–2020	UNFPA	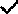	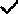	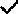	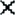	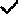
Good practices in SSTC for sustainable development- Volume 3^23^											
10.	The Mobile services strategy (report/case study) (23a).	Chad, Mali, Mauritania, Niger, Dijibouti, Tunisia	Report (case study)	2020	N-2005–2008 M-2007–2011	UNOSCC	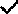	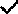	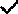	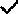	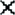
11.	Improvement of maternal and child health on the Northern border of the Dominican Republic and Haiti (case study) (23b).	Dominican Republic, Haiti	Report (case study)	2020	2018–2020	UNOSCC	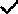	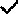	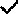	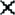	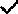
Good practices in SSTC for sustainable development-Volume 2^24^											
12.	Promoting FP (case study) (24a).	Afghanistan, Algeria, Azerbaijan, Bangladesh, Chad, Ethiopia, Ghana, Guinea, India, Malaysia, Maldives, Mali, Nepal, Niger, Nigeria, Pakistan, Philippines, Sri Lanka, Sudan, Indonesia	Report (case study)	2018	2013–2020	UNOSCC	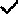	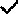	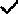	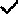	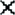
13.	Indonesia’s Maternal and Child health handbook (case study) (24b).	Afghanistan, Kenya, Philippines, Tajikistan, Thailand Indonesia	Report (case study)	2018	2017–2019	UNOSCC	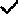	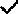	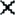	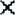	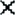
Annual report of Indonesia’s SSTC^25^											
14.	Training course on empowering women through socioeconomic and cultural intervention (25a).	Bhutan, Fiji, Iran, Malaysia, Maldives, Myanmar, Nepal, Pakistan, Philippines, Sri Lanka, Vietnam, Indonesia	Report (case study)	2014	2014 (21–30 September)	National coordination team of SSTC	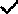	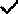	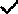	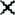	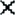
15.	The training course on Population, FP and family development (25b).	Vietnam, Indonesia	Report (case study)	2014	2014 (26–30 October)	National coordination team of SSTC	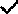	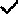	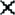	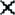	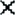
16.	What five Sahel countries learnt from Bangladesh about strengthening population policies.^13^	Sahel countries (Burkina Faso, Chad, Mali, Mauritania, Niger), Bangladesh	Press release	2016 (June 23)	7 days	World bank	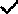	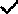	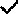	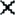	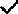
17.	Training on comprehensive Right based in FP services (Centre of Excellence).^26^	Bangladesh, Ghana, Papua New Guinea, Timor-Leste, Indonesia	Press release	2020	20 days (September 3–22 2018)	BKKBN	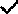	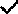	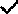	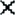	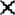
18.	Training on strategic partnership with Muslim religious leaders in FP.^27^	Azerbaijan, Chad, Nepal, Niger, Ghana, Guinea, Malaysia, Mali, Sri Lanka, Indonesia	Press release	2020	23–28 April 2018	BKKBN	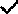	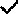	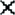	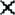	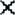
19.	Training course on public private partnership on FP services interventions.^28^	Pakistan, Indonesia	Press release	2020	16–20 April 2018	BKKBN	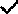	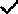	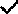	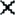	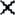
20.	SSTC Online training on strategic partnership with Muslim religious leaders in reproductive health, FP, prevention of VAW and child marriage.^14^	Bangladesh, Pakistan, Nepal, Philippines, Indonesia	Press release	2021	24–25 May 2021	BKKBN	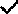	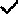	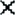	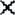	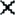
21.	BKKBN, Ministry of State Secretariat and UNFPA hosted a webinar on strategic partnership with Muslim religious leaders in FP for Malaysian delegates.^15^	Malaysia, Indonesia	Press release	2020	10 September 2020 (3 hours)	BKKBN	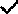	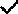	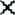	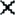	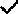
22.	Increasing use of postpartum FP and the postpartum IUD: Early experiences in West and Central Africa.^7^	Benin, Chad, Cote d’Ivoire, Niger, Senegal, Guinea	Manuscript	2016	November 2013	Global health; Science and practice	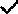	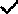	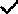	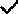	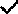
23.	Umbrella to Accelerator: WHO-FP Accelerator project newsletter (issue 4).^8^	Nepal, Sri Lanka, Nigeria, Uganda	Newsletter	Sept 2020	N.A.	FP2020	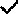	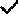	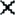	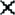	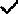
24.	Responsible Parenthood and Reproductive Health Act of 2012 Annual report.^17^	Philippines, Indonesia	Report (case study)	2020	2011	Department of Health, Philippines	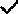	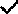	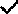	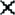	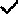
25.	Enhancing SSTC (Kollo project).^16^	Tunisia, Niger	Report (case study)	2009	2001–2004	UNDP	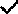	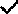	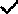	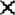	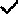
26.	Community based access to injectable contraception-An emerging standard of practice.^11^	Kenya, Nigeria, Rwanda, Uganda	Press release	2013	2008–2013	FHI 360	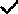	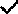	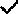	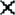	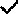
27.	Formative evaluation of UNFPA approach to SSTC.^12^	Syria Arab Republic, Islamic Republic of Iran	Report (case study)	2020	2017	UNFPA	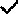	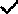	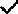	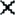	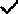
28.	Improving access to quality FP services in Nepal and Sri Lanka: insights from a SSLE.^9^	Nepal, Sri Lanka	Manuscript	2022	2019–2022	BMJ Global Health	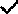	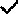	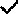	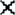	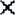
Good practices in SSTC for sustainable development-Volume 4^10^											
29.	FP Accelerator project^10^	Nepal, Sri Lanka	Report (case study)	2022	2019–2022	UNOSCC	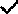	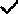	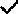	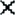	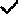

Purpose of SSLE in FP.

FP, family planning; N.A., not available; SSLE, South-South learning exchange; UNDP, United Nations Development Programme; UNFPA, United Nations Population Fund; UNOSCC, United Nations Office of South-South cooperation.

### Review findings

All articles (100%) reported the purpose, 28 (97%) reported the approaches, 22 (76%) reported outputs, 7 (21%) reported outcomes, 12 (41%) described the enablers and 1 (3%) reported the barriers of SSLE.

The purposes identified from the included articles were learning new service delivery skills to improve access to quality FP services, advocating for a policy or programme change and building the capacity of FP providers, policy-makers and the community. One article reported more than one purpose of the SSLE (18b). Table 3 summarises the studies.

**Table 3:** Summary of studies reporting the purpose of SSLE in family planning

S. no	Purpose of SSLE in family planning	No of studies	Reference
1.	Learn new service delivery skills to improve access to quality FP services	9	7, 8, 9, 10, 11, 16, 18d, 22c, 23a
2.	Build capacity of FP providers, policy-makers and community	19	12, 13, 14, 15, 17,18a, 18b, 18c, 18e, 22a, 22b, 23b, 24a, 24b, 25a, 25b, 26, 27, 28
3.	Advocate for a policy or programme change	2	18b, 18f

FP, family planning; SSLE, South-South learning exchange.

#### Service delivery (learn new service delivery skills to improve access to quality FP services)

Nine studies (7, 8, 9, 10, 11, 16, 18d, 22c, 23a) reported learning a new service delivery skill as the purpose of SSLE in FP. Retired Fijian midwives were deployed to Samoa during the measles outbreak to support the continuity of FP services (22c). Tunisia shared its experiences and lessons from setting up mobile health teams with Niger, following which four mobile health teams were set up in Niger to increase access and awareness of reproductive health (RH) and FP services (16, 23a). Health providers from Benin, Chad, Cote d’Ivoire, Niger and Senegal were trained in Guinea on PPFP/PPIUD (postpartum intrauterine device) to facilitate scaling up these services at the facility level in their respective countries.^7^ The reciprocal exchange between Nepal and Sri Lanka focused on Nepal learning about the integration of FP counselling and services using a life course approach to improve PPFP uptake and Sri Lanka learning about establishing a web-based e-logistics management information system (eLMIS) for contraceptives to enable district and central levels commodity security.^8–10^ Niger shared its experiences and learnings on Schools of Husbands, established to promote women’s access to RH information and services with other African countries (Burkina Faso, Cameroon, Cote d’Ivoire, Guinea, Mali, Mauritania, Senegal, Sierra Leone, Togo) (18d). Officials from Rwanda, Kenya and Nigeria visited Uganda to learn about scaling up community-based access to injectable contraceptives.^11^ In summary, SSLE has been used to improve FP service delivery by learning new skills like scaling up PPIUD services in facilities, integrating PPFP services in ANC care, setting up eLMIS, scaling up the use of community-based injectables and educating men on the importance of FP.

#### Capacity building of FP providers, policy-makers and community

Nineteen studies (12, 13, 14, 15, 17,18a, 18b, 18c, 18e, 22a, 22b, 23b, 24a, 24b, 25a, 25b, 26, 27, 28) reported capacity building as the purpose of the SSLE. Of these, 10 studies (14, 15, 17, 18a, 18b, 18c, 22b, 24a, 27, 28) reported capacity building of Muslim religious and traditional leaders on FP in Indonesia. Six studies (18e, 22a, 24b, 25a, 25b, 26) reported conducting FP training programmes through the Centre of excellence in Indonesia and the Mauritius Institute of Health. One study reported capacity building of midwives on RH management, FP, monitoring and evaluation.^12^

Five Sahel countries (Burkina Faso, Chad, Mali, Mauritania, Niger) learnt about population policy and how to sustain support and implement changes to increase the use of RH and FP services.^13^ One study aimed to develop community interventions to inform and motivate women on FP services (23b). Thus, SSLE was used to build the capacity of Muslim religious leaders (MRLs), health providers, midwives and the community on the benefits of FP services.

#### Advocacy for a policy or programme change to introduce a new practice

Two studies (18b,18f) reported advocacy for policy/programme change as the purpose for SSLE. Sudan embarked on an SSLE with Morocco to build its midwifery education programme and services (18f). Indonesia learnt from the Philippines about decentralising FP programmes to local governments and the role of female religious leaders in FP promotion (18b).

### Approaches used for conducting SSLE

From this review, the approaches used in conducting an SSLE were classified into four categories: study tours (reciprocal and non-reciprocal study tours) exchange, virtual exchange, expert visits and mixed methods. Table 4 summarises the studies.

**Table 4:** Summary of studies reporting approaches used to conduct SSLE in family planning

S. no	Approaches used for conducting SSLE	No of studies	Reference
1.	Study tour	16	
	(1) Non-reciprocal study tours	13	12, 13, 18a, 18c, 18d, 18f, 22a, 24b, 25a, 25b, 26, 27, 28
	(2) Reciprocal exchange	3	17, 18b, 23b
2.	Virtual exchange	3	14, 15, 18e
3.	Expert visit	3	16, 22c, 23a
4.	Mixed method	6	
	(1) Study tour and expert visits	3	7, 22b, 24a
	(2) Virtual reciprocal exchange	3	8,9,10

SSLE, South-South learning exchange.

#### Study tours

##### Non-reciprocal study tours

Thirteen studies reported using study tours for conducting the learning exchanges (12, 13, 18a, 18c, 18d, 18f, 22a, 24b, 25a, 25b, 26, 27, 28). A combination of one or more of the following activities was used in the study tours: training workshops, class discussions/lectures, field visits or internships. Training workshops were the most common activity lasting for a few days to weeks in the host country.

##### Reciprocal study tours

Three studies reported using reciprocal/bilateral study tours between the two countries (17, 18b, 23b). Two studies used a mix of activities like training workshops, field visits, internships and exchange visits (17, 18b). In another study, joint meetings were conducted between health teams from both countries to share good practices at three levels of healthcare and with community leaders and community health workers at the community level from both countries to ensure ownership (23b).

#### Virtual exchanges

Three studies reported using virtual exchanges to conduct SSLE (14, 15, 18e). Due to the COVID-19 pandemic, the annual training workshop in Indonesia on strategic partnership with MRLs in FP was held virtually,^14^ and a webinar was organised by Indonesia for Malaysian delegates.^15^ One study used distance learning courses focusing on FP and maternal health for the learning exchange (18e). Thus, most of the virtual exchanges were reported after the COVID-19 pandemic.

#### Expert visits

Three studies reported using expert visits for conducting SSLE (16, 22c, 23a). A RH expert from Tunisia set up a new strategy and approach in service provision, IEC and management of activities in Niger (16, 23a). Retired midwives from Fiji were deployed to Samoa to support the overburdened health staff and midwives to continue providing MCH and FP services during the measles outbreak (22c). Thus, FP experts were deployed to the knowledge-seeking countries to share their knowledge and expertise in improving FP services.

#### Mixed methods

Six studies reported using more than one method to conduct SSLE (7, 8, 9, 10, 22b, 24a). Of these, three studies used a study tour with expert visits (7, 22b, 24a) and three studies reported using reciprocal and virtual exchanges.^8–10^ In Chad and Niger, following the study tour, Indonesian Muslim leaders were invited to facilitate the workshop on FP and Islam (24a). Similarly, in Nepal, Indonesian MRLs were invited to facilitate the first-ever national-level workshop on FP, following the study tour (22b). In another study, following the study tour, the trainers visited the participants in their hospitals to monitor the new skills and services and help providers address challenges and manage service delivery.^7^ WHO supported reciprocal virtual learning exchanges between Nepal-Sri Lanka and Nigeria-Uganda.^8–10^ The most common combination for conducting an SSLE is when participants from a knowledge-seeking country conduct a study tour, followed by a visit of an FP expert to their country to follow-up on the learnings. The other combination is that participants from both countries learn from each other through study tours and virtual exchanges.

### Key outputs from SSLE

From our review, the FP outputs achieved from SSLE are grouped into five categories: training of health professionals, policy dialogue, service delivery, advocacy/awareness-raising campaigns and action plans/FP projects. A few studies reported more than one FP output from the SSLE. Table 5 summarises the studies.

**Table 5:** Summary of studies reporting key outputs from the SSLE

S. no	Key outputs from SSLE	No of studies	Reference
1.	Training of health professionals	6	7, 11, 12, 18e, 22a, 23b,
2.	Policy dialogue	10	9, 10, 16, 17, 18a, 18b, 18c, 18f, 22b, 24a
3.	Service delivery	3	7, 18c, 22c
4.	Advocacy/awareness raising campaigns	6	9, 10, 18a, 18c, 22b, 24a
5.	Plans and project initiation	9	13, 16, 18a, 18f, 22b, 23a, 25a, 26, 28

SSLE, South-South learning exchange.

#### Training of health professionals

Six studies reported training of health professionals as the FP output (7, 11, 12, 18e, 22a, 23b). Training of trainer’s course on RH, with a focus on FP held at the Mauritius Institute of Health, resulted in 1500 trained health personnel from African countries (18e). Five batches of training were conducted between 2015 and 2019 at the Centre of Excellence, Indonesia, for 39 medical doctors and Ob-Gyns from 10 countries in Asia and Africa (22a). One of the participants from Ghana cascaded training to 550 health professionals (22a). In the reciprocal exchange between the Dominican Republic and Haiti, 199 community health workers (between 2018 and 2020) were trained on priority topics, including FP (23b). Sixteen Syrian midwives were trained in Iran on RH management, FP, monitoring and evaluation in 2017.^12^

Twenty-one providers from 5 countries (Benin, Chad, Cote d’Ivoire, Niger, Senegal) were trained in PPFP counselling, of which 18 were also trained in PPIUD insertion. In addition, 13 trainers were updated on PPFP and 46 providers were trained in PPFP counselling, of which 33 were trained on PPIUD insertion in Togo.^7^ Officials from Kenya, Nigeria and Rwanda visited Uganda to learn from the country’s experience in scaling-up community-based access to injectables.^11^

#### Policy dialogue

Ten studies reported policy dialogue as the key FP output (9, 10, 16, 17,18a, 18b, 18c, 18f, 22b, 24a). Of these, six studies reported that a fatwa/declaration supporting the use of FP among the Muslim community was passed by the Philippines, Guinea, Chad and Nepal (17,18a, 18b, 18c, 22b, 24a). Following the SSLE with Indonesia, the Philippines endorsed a fatwa (official ruling) on the family model in Islam and that FP was the responsibility of both husband and wife (17,18a). In Guinea, MRLs and Christian religious leaders produced a declaration supporting the national FP programme (18a, 24a). The main Muslim political entity in the Philippines accepted RH/FP as a development issue and implemented the FP programme in five pilot municipalities in the Autonomous Regions of Muslim Mindanao region, and a new fatwa was passed in 2015, clarifying that FP is not forbidden in Islam (18b, 24a). In Chad, a ‘declaration of N’Djamena’ was issued, which stated that the spacing of births is prescribed by the Quran (18c). The MRLs from Kapilvastu district in Nepal launched a ‘Khusahal Parivar Approach/ Happy family approach’, a five-point declaration to promote family well-being within their community (22b).

In Sudan, the community midwife education programme was discontinued, followed by a gap analysis to identify specific areas that needed improvement, resulting in the development of a new midwifery programme launched in 2018 in four schools and two universities that started a midwifery diploma course (18f). In Niger, the Ministry of Health integrated the strategy of mobile units into the National health development plan 2006–2009 as it proved very effective in providing services for excluded populations.^16^

Three indicators to monitor PPFP uptake were proposed for inclusion in Demographic Health Survey and National Health Facility Survey in Nepal. The Ministry of Health had allocated funds to all provinces to initiate and strengthen PPFP in hospitals.^9 10^ Sri Lanka had introduced a dedicated page on the reporting system of contraceptive commodities in its District Health Information Software 2.^9 10^

#### Service delivery

Three studies reported service delivery as the FP output (7, 18c, 22c). Following the SSLE, an FP unit was set up within the health centre located inside the main mosque of N’Djamena, Chad (18c). The Fijian midwives provided FP services for 276 clients during the measles outbreak in Samoa (22c). Following the learning visit to Guinea, there was an increase in the number of sites (from 4 in 2014 to 19 in 2016) providing PPFP and PPIUD services in 6 countries (Benin, Chad, Cote d’Ivoire, Niger, Togo, Senegal), with more than 15 000 women being counselled on PPFP services.^7^

#### Advocacy/awareness-raising campaigns

Six studies reported advocacy and awareness-raising campaigns as key outputs (9, 10, 18a, 18c, 22b, 24a). Following the training in Indonesia, Nepal conducted a district-level orientation on FP and Islam through a discussion with Ulemas (religious scholars), madrasas (Islamic schools) and parents of Muslim girls to disseminate FP-related messages, while Chad focused on behaviour change communication intervention through a network of partner agencies (18a).

The Higher Council of Islamic Affairs organised a Forum on ‘Islam, family well-being and demographic dividend’ attended by 2000 religious leaders and technical experts from various regions of Chad and 20 other countries of West and Central Africa, the Arab States and Asia (18c). Following the forum, awareness-raising campaigns on the benefits of FP were held using radio broadcast networks, and Imams delivered messages on the benefits of FP in mosques during Friday prayers in N’djamena, Chad (18c). MRLs in five municipalities of ARMM are promoting FP through local radio stations (24a).

A book developed by UNFPA Indonesia and BKKBN on ‘FP, RH and gender: Islamic perspective’ was translated into Nepali language and is used by MRLs for community level orientation (22b, 24a). Nepal developed a PPFP advocacy tool that was used to conduct policy dialogue with policy-makers and programme managers in two provinces.^9 10^

#### Plans and project initiation

Nine studies reported that action plans were developed, and FP projects were initiated following the learning exchanges (13, 16, 18a, 18f, 22b, 23a, 25a, 26, 28). Following the exchange with Indonesia, the action plan developed with Nepal was converted into a 4-year project funded by DFID that reaches out to Ulemas, men and women from Muslim communities to disseminate FP-related messages (22b). Following the success of the mobile health clinics in the Kollo district, the Niger Board of FP received a UNDP award, which motivated the World Bank, UNFPA and Japan International Cooperation Agency (JICA) to support the promotion of FP/SRH services in three more areas of Niger (Niamey, Dosso, Zinder) (23a). Similarly, the success of the Kollo project was replicated in Chad and Mauritania, with financial support from the World Bank and Spanish cooperation agency and technical assistance from the Tunisian Board of FP.^16^

At the end of the study tour, seven studies reported that participants developed and presented an action plan at the training workshops (13, 18a, 18f, 22b, 25a, 26, 28).

### FP outcomes

Seven studies report the FP outcomes, with some reporting more than one outcome. The most frequently reported outcome is the contraceptive prevalence rate, followed by the quality of FP services and the unmet need for FP.

#### Contraceptive prevalence rate

Seven studies reported improved contraceptive uptake following the learning exchanges (7, 16, 18b, 18c, 22b, 23a, 24a). A slow increase in the contraceptive prevalence rate following the declaration by religious leaders supporting the national FP programme was noted in Guinea (24a). In Chad, the FP unit set up at the main mosque of N’Djamena resulted in approximately 1000 new acceptors of modern contraceptive methods per year (18c). The learning exchange between Nepal and Indonesia resulted in a twofold increase in the uptake of FP among the Muslim communities in Nepal (22b). Following the establishment of mobile clinics, there was an increase in CPR from 1.5% in 2000 to 22.5% in 2005 in Niger and an improved uptake of contraceptives by 118% of set targets in Chad (23a). The learning exchange with Guinea resulted in 2269 new acceptors of PPIUD in five countries (Benin, Niger, Senegal, Chad and Cote d’Ivoire).^7^ Following learnings from Tunisian expert, the mobile health clinics in Kollo, Niger had a 21% increase in contraceptive prevalence^16^

#### Unmet need for FP

One study reported a decline in the unmet need for FP (18b). The learning exchange between Indonesia and the Philippines resulted in an increase in the use of modern contraceptives and a decline in the unmet need for FP (18b).

#### Quality of FP services

Two studies reported improved quality of FP services. The mobile health units in Niger improved coverage of the population by quality RH/FP services from 27% in 2000 to 80% in 2005 (23a). Following the training visit to Guinea, there was an improved quality of PPFP services in the five Francophone countries.^7^

### Enablers and barriers

Twelve studies reported the enablers (7, 9, 10, 12, 13, 16, 17, 18a, 18b, 18c, 18f, 23b), and only one study^9^ reported on the barriers to SSLE.

#### Enablers

Six studies (7,9, 18f, 23b, 13, 17) reported that the learning exchange was successful because both countries had similar cultural and religious backgrounds. Seven studies (9, 12, 16, 18a, 18b, 18c, 18f) reported engaging religious leaders and high-level administrators from the start of the learning exchange as an enabling factor. Four studies^7 9 10 16^ reported that country ownership during planning, implementation and documentation of the SSLE determines the success of the learning exchange. Five studies^9 10 12 16 17^ reported that the need and purpose of the learning exchange should be identified by the Ministry of Health and other stakeholders based on ground-level realities for it to be successful. One study reported that dissemination of positive programme experiences at conferences led to the expansion of the initiative and the importance of careful record keeping, regular monitoring and feedback to improve the quality of the learning exchange.^7^

#### Barriers

One study^9^ reported that learning exchanges are time-consuming as many meetings are needed between countries and within countries to complete the process of sharing knowledge leading to the implementation of the learnings.

### Stakeholder consultations

Sixteen experts from PPD (n=3), UNFPA (n=4), Blue Ventures (n=2), BKKBN, Knowledge Success, Rising outcomes, FHI 360, Hero, WHO and Nahdlatul Ulama with previous experience conducting SSLEs, were interviewed. Half of the respondents were male, and half were female. The experts were a part of SSLEs between different countries and were involved either as implementing partners or facilitators.

The purpose of SSLE reported by a majority of experts was capacity building of providers, policy-makers and the community, and study tours were the most commonly reported method to conduct SSLEs, both consistent with the literature. ‘Using a cascade training model, 1200 grass roots religious leaders across the four countries benefited from training in RH and rights. This programme ultimately contributed to improved health and rights for women in Muslim communities through the removal of prior barriers to the use of FP and the promotion of modern contraceptive’.

The key FP outcomes that could be attributed to SSLE were an increase in contraceptive prevalence rate and a reduction in unmet needs for FP. ‘In one facility at the Mosque, we saw an increase in women’s acceptance of FP contraceptive than before’. However, challenges in quantifying this improvement remain, as mentioned in an included paper.^18^ Attributing an increase in the FP uptake to the learning exchange or other confounding factors is difficult.

The experts were enthusiastic about sharing their experience of SSLE and perceived participant selection and a country-driven approach as critical elements for a successful exchange. Some participants ‘do not really care about the purpose of exchange. They never took the initiative to take the next action’. For this reason, ‘part of the success of the SSLE comes from individuals, their connection and passion around it’. This highlights the crucial role of champions and trusted stakeholders within the country.

Experts highlighted the positive impact of conducting an SSLE between countries with similarities in culture, religion, geography, etc and the importance of basing it on a needs assessment ‘Identifying the needs and having a clear baseline from the beginning of the process is the most important and difficult part of SSLE’ and having in-country champions that drive the learning exchange in partnership with trusted stakeholders. They recognised the importance of having a good facilitator as ‘Discussions can be quite sensitive, bring ideas and solutions, but can cause conflicts between teams, so we need someone to steer around these difficulties’.

Tracking progress, follow-up after completion and documentation were perceived as scarce; this highlights that tools for monitoring and documenting are vital to achieving the final goal. ‘The follow-up is often not done. Generally, this depends on budget’.

Some of the barriers mentioned were that the SSLE is a costly and demanding process requiring time to schedule and conduct meetings, carry out advocacy activities and plan study tours. Finally, experts reported the existence of scarce policies and strategies at the national and international levels that regulate and manage the SSLEs ‘Policy-makers have scarce knowledge and skills to conduct SSLE’.

table 6 summarizes the enablers and barriers to conducting a SSLE, based on findings from the literature and stakeholder consultations.

**Table 6:** Summary of enablers and barriers to conducting SSLEs based on included studies and stakeholder consultations

Enablers	Barriers
Engaging high-level administrators and religious leaders from the start of the SSLE. Countries with similar cultural and religious backgrounds Country-led need and process for SSLE Purpose based on a needs assessment Country ownership during planning, implementation and documentation Dissemination of positive programme experiences Presence of in-country champions that can drive the learning exchange Good facilitator	Time-consuming process for both countries Scarce policies and strategies to regulate and manage SSLEs Costly and demanding process

SSLE, South-South learning exchange.

## Discussion

This is the first scoping review that systematically identified and summarised the studies on SSLE in FP. This scoping review aimed to collate the evidence available on purpose, approaches, outcomes, outputs, enablers and barriers on using SSLE in FP. The methods used throughout the different stages of the review were rigorous and transparent, and the process is documented in sufficient detail to replicate the research approach. Additionally, 16 experts from organisations with previous experience conducting SSLEs were interviewed. Thus, the information gathered from the literature was enriched with insight from the experts directly involved in the process.

The most frequently reported outcome was the contraceptive prevalence rate (24% of all studies). In comparison, a few studies reported improvement in the quality of care in FP (7%) and a reduction in the unmet need for FP services (3.4%). No studies reported other important outcomes, such as equitable access to FP services (same access to information and quality services), access to a broad modern method mix, increasing autonomy in contraceptive decision-making, and contraceptive method continuation, discontinuation and switching.^19–21^ Although FP outcomes were reported by a few studies, there is insufficient evidence to determine the extent to which SSLE has contributed to FP outcomes. It is difficult to present a general effect estimate for the best approaches or the extent to which SSLE contributed to FP outcomes because of multiple factors. Included studies did not elaborate on the details of the process of SSLE and the role/involvement of stakeholders, nor did the studies include information on follow-up after the learning exchange is completed or information on baseline data.

Approximately half (45%) of the studies on outputs from SSLE reported a policy dialogue. But there was insufficient information on the implementation of the learnings or the documentation of the lessons learnt. Study tours were identified as the most common approach (57%) for conducting SSLE among all studies reporting approaches, which was also validated by expert interviews. However, the included studies do not provide information on how the purpose was identified and how the participants were selected for the study tour, nor are any details provided on whether a follow-up or an evaluation of the skills was done. The experts identified SSLE as an important process for learning new knowledge, but highlighted the need for a standard methodology to conduct, implement and document learnings from these exchanges.

UNFPA evaluated the SSC^12^ and shared some enablers and lessons learnt. It reported that learning exchanges are one of the best modalities for addressing several SRHR issues, where learning between similar cultural attitudes is much more effective than from Global North countries. Follow-up of the SSC exchanges at the country level was reported as a major challenge in enhancing SSC effectiveness within UNFPA. Evaluating SSC is a major area that needs improvement, as suggested during our stakeholder consultations. Less than half of the survey respondents reported that documenting SSC lessons and results are inadequate. This absence of coherent and consistent documentation and systemised information is a major stumbling block for facilitating and communicating learning exchanges and institutional memory and learning.^12^ These findings are similar to the enablers and barriers listed in our review.

We are uncertain about the effectiveness of SSLE for addressing SRHR issues, including access to FP because the evidence was very low certainty. Only a few studies report the FP outcomes following an SSLE. Only two studies included in the review were peer-reviewed publications on SSLE in FP. Most of the included studies were reports, case studies or press releases. The limited publications demonstrate the need to emphasise and support documentation of the approaches used and the lessons learnt using SSLE.

## Conclusions

Ours is the first scoping review to explore the purpose, approaches, enablers, barriers and outcomes of SSLE in FP. SSLE probably improves knowledge sharing among programme managers and policy-makers and supports them in making informed decisions to scale up evidence-based practices in FP.

We are uncertain about the effectiveness of the approaches used for SSLE, the enablers and barriers and the outcomes achieved because of the lack of high-quality published data related to SSLE. Stakeholders conducting SSLE must document their experiences in detail, including the outcomes achieved. Further studies are needed to identify the best approach to conduct SSLE to improve access to right-based equitable FP services.

## Data Availability

All data relevant to the study are included in the article or uploaded as online supplemental information.
