# Supplementary material for: Scoping review on the use of South-South learning exchange to scale up evidence-based practices in family planning
Source: BMJ Glob Health. 2023 Jun 12;8(6):e011635. doi: 10.1136/bmjgh-2022-011635 (PMC10277101; doi:10.1136/bmjgh-2022-011635)
Supplement: Supplementary data [file bmjgh-2022-011635supp002.pdf]

Supplementary material 2: Search strategy for South-South learning exchange in family planning: scoping review

#	Database : PubMed<br><br>Database Provider : http://www.pubmed.gov<br><br>Date limits : Language limits :<br>Other notes on the search :	Results
	("South-South" OR ("International Cooperation"[MH] AND "Developing Countries"[MH])) AND ("learning exchange" OR " knowledge exchange" OR "Peer to peer " OR "cooperation" OR "information sharing" OR "information exchange" OR "knowledge sharing" OR "knowledge exchange" OR "learning exchange") AND (reproduct* OR "family planning" OR contracept* OR "Family Planning Services" OR "Reproductive Medicine" OR "Population Control" OR "Population Growth" OR Contraception OR Fertility OR "Contraception Behavior" OR "Embryo Transfer" OR "Intrauterine Devices" OR "Long Acting Reversible Contraception" OR "Maternal Child Health Centers" OR "Pregnancy In Adolescence" OR "Reproductive Techniques Assisted")	538
#	Database : EMBASE<br><br>Database Provider : http://www.embase.com<br>Date limits : none<br>Language limits :<br>Other notes on the search :	Results
	('South-South' OR ('developing country'/exp AND 'international cooperation'/exp/mj)) AND ('learning exchange' OR ' knowledge exchange' OR 'Peer to peer ' OR 'cooperation' OR 'information sharing' OR 'information exchange' OR 'knowledge sharing' OR 'knowledge exchange' OR 'learning exchange') AND (reproduct* OR 'family planning' OR contracept* OR 'Family Planning Services' OR 'Reproductive Medicine' OR 'Population Control' OR 'Population Growth' OR Contraception OR Fertility OR 'Contraception Behavior' OR 'Embryo Transfer' OR 'Intrauterine Devices' OR 'Long Acting Reversible Contraception' OR 'Maternal Child Health Centers' OR 'Pregnancy In Adolescence' OR 'Reproductive Techniques Assisted')	607

#	Database: Global Index Medicus (GIM)<br><br>Database Provider : http://www.globalhealthlibrary.net/<br><br>Date limits : none<br>Language limits : none<br>Other notes on the search : Filter activated to limit to Regional Databases (AIM, LILACS, IMEMR, IMSEAR & WPRIM)	Results
	("South-South" OR south*south OR south?south OR (mh:("International Cooperation") AND mh:("Developing Countries"))) AND (reproduct* OR "family planning" OR contracept* OR "Family Planning Services" OR "Reproductive Medicine" OR "Population Control" OR "Population Growth" OR Contraception OR Fertility OR "Contraception Behavior" OR "Embryo Transfer" OR "Intrauterine Devices" OR "Long Acting Reversible Contraception" OR "Maternal Child Health Centers" OR "Pregnancy In Adolescence" OR "Reproductive Techniques Assisted")	79
#	Database: CINAHL<br><br>Database Provider: http://www.ebsco.com<br>Date limits: none<br>Language limits: none<br>Other notes on the search:	Results
	("South-South" OR ("International Cooperation" AND "Developing Countries")) AND (reproduct* OR "family planning" OR contracept* OR "Family Planning Services" OR "Reproductive Medicine" OR "Population Control" OR "Population Growth" OR Contraception OR Fertility OR "Contraception Behavior" OR "Embryo Transfer" OR "Intrauterine Devices" OR "Long Acting Reversible Contraception" OR "Maternal Child Health Centers" OR "Pregnancy In Adolescence" OR "Reproductive Techniques Assisted")	22
#	Database: HINARI<br>Database Provider: https://www.who.int/hinari/en/<br><br>Date limits: none<br>Language limits: none<br>Other notes on the search: Search beyond your collection	Results

	Limit excluded newspaper articles	
	(TitleCombined:("south to south" OR "south south")) AND (("family planning" OR contracept* OR "Reproductive Medicine" OR "Population Control" OR "Population Growth" OR Contraception OR "Maternal Child Health Centers" OR "Pregnancy In Adolescence" OR "birth control"))	45
#	Database: Web of Science<br><br>Database Provider: https://www.webofscience.com/wos/woscc/<br><br>Date limits: none<br>Language limits: none<br>Other notes on the search:	Results
	(ALL="South-South" OR (TS="International Cooperation" AND TS="Developing Countries")) AND ALL=(reproduct* OR "family planning" OR contracept* OR "Family Planning Services" OR "Reproductive Medicine" OR "Population Control" OR "Population Growth" OR Contraception OR Fertility OR "Contraception Behavior" OR "Embryo Transfer" OR "Intrauterine Devices" OR "Long Acting Reversible Contraception" OR "Maternal Child Health Centers" OR "Pregnancy In Adolescence" OR "Reproductive Techniques Assisted")	78
#	Database: WorldCat<br>Database Provider: https://www.worldcat.org/<br>Date limits: none<br>Language limits: none<br>Other notes on the search: Non journal articles	Results
	'ti:("South-South" OR ("International Cooperation" AND "Developing Countries")) AND (reproduct* OR "family planning" OR contracept* OR "Family Planning Services" OR "Reproductive Medicine" OR "Population Control" OR "Population Growth" OR Contraception OR Fertility OR "Contraception Behavior" OR "Embryo Transfer" OR "Intrauterine Devices" OR "Long Acting Reversible Contraception" OR "Maternal Child Health Centers" OR "Pregnancy In Adolescence" OR "Reproductive Techniques Assisted")'	92

Allagh KP, *et al. BMJ Global Health* 2023; 8:e011635. doi: 10.1136/bmjgh-2022-011635
